# Physiological characteristics and green production NiO nanoparticle synthesis employing Moringa Oleifera lam extract to assess in vitro cytotoxicity, antibacterial

**DOI:** 10.1038/s41598-025-07796-8

**Published:** 2025-09-30

**Authors:** Abdulhassan Mahdi Salih, Zainab Mohammed Ali Hassan, Entidhar Jasim Khamees, Olcay Gençyılmaz

**Affiliations:** 1 Department of Medical Physiology, College of Medicine, University of Dhi Qar, Nasiriyah, Iraq; 2 Department of Family and Community Medicine, College of Medicine, University of Alayen lraq, Nasiriyah, Iraq; 3 Department of Physiology and Medical Physics, College of Medicine, University of Babylon, Hillah, Iraq; 4 Department of Material and Material Processing Technologies, Physics, Çankırı Karatekin University, Çankırı, Turkey

**Keywords:** Moringa Oleifera, NiO, HEP-G2, Antibacterial activity, Antibiotic, Cytotoxicity, Anticancer, Physiology, Nanoscience and technology, Optical physics

## Abstract

Moringa Oleifera is a medicinal plant that is used in the production of cytotoxic agents, which are used in cancer treatment to prevent the division and spread of cancer cells. The present study investigates the cytotoxic activity of NiO nanoparticles (NiONPs) produced by means of green synthesis using Moringa Oleifera on human cancer cell lines (HEP-G2). The antibacterial activity of these nanoparticles was also evaluated in relation to the carbapenem (CRO), chloramphenicol (C), ampicillin (AM), cephalothin (KF) and meropenem (MEM) antibiotics, as well as Gram-positive Streptococcus mutans (*S. mutans*) (+) and Gram-negative Escherichia coli (*E. coli*) (-) bacteria. NiONPs demonstrated a high cytotoxic value of 91.05% against HEP-G2 cancer cells. The study established that 1 µg/ml resulted in 85.2% of HEP-G2 cancer cell death. The study demonstrated high levels of antibacterial resistance at low concentrations (0.0315 µg/mol). The high sensitivity of the bacteria was determined for Chloramphenicol (C) and Meropenem (MEM) for *S. mutans* and for all antibiotics for *E. coli* bacteria. It was demonstrated that *S. mutans* and *E. coli* bacteria exhibited high susceptibility to only chloramphenicol (C) and meropenem (MEM) antibiotics. The NiONPs demonstrated a spherical morphology, with grain sizes ranging from 24 to 40 nm, and a surface plasma resonance (SPR) peak at 356 nm. NiONPs prepared with Moringa oleifera plant are suitable for biomedical applications due to their very high level of toxicity against HEP-G2, strong antibiotic sensitivity and antimicrobial activity against S. mutans and E. coli.

## Introduction

The emergence of nanotechnology has recently made it one of the most important and interesting fields in physics, chemistry, engineering and biology^1^. This field offers immense potential for numerous discoveries that will determine the course of technological growth in a wide range of applications^2^. Nanotechnology research encompasses a wide range of disciplines, including catalytic nanoparticle chemistry and quantum laser physics. The creation of new substances with unique physical, chemical and biological properties, such as nanotubes, nanorods and nanoparticles, is a key area of research^39,40^. The practical applications of nanotechnology have been demonstrated in a variety of fields, including microprocessor technology, medical research and scientific research^3,4,23^. The field of nanotechnology is undergoing rapid development and expansion, with a growing number of applications in various sectors^5^. In recent years, there has been a shift towards biological synthesis as an alternative to physical and chemical synthesis processes, which have been shown to produce undesirable results^6^. These biological synthetic methods are currently being used to identify novel applications for nanoparticles derived from plant extracts^7–9,17^.

A wide range of synthetic methods for the production of nanoparticles have been developed by researchers, providing significant benefits to ecosystems and biodiversity through processes that are non-toxic, non-polluting and environmentally friendly, including bacteria, fungi and plants^10,11^. Nevertheless, a significant number of nanoparticles have been shown to have adverse effects at the nanoscale. Consequently, there is an increasing focus on environmentally friendly alternatives that use natural resources such as plants and microorganisms to mitigate toxicity^12,13^.

The green synthesis of NiO nanoparticles utilizing Moringa Oleifera Lam as a pharmaceutical agent has been shown to be both environmentally benign and economically effective^10,14^. This plant is widely used in nutrition and has a number of pharmacological actions, including anti-diabetic, protective effects on the liver, anti-inflammatory, anti-fertility, anti-cancer, anti-microbial, antioxidant, cardiovascular, anti-ulcer, CNS activity, anti-allergic, wound healing, analgesic, and antipyretic properties^15^.

Chemical production of various nanoparticles such as TiO_2_, SiO_2_, ZnO, CuO, NiO and others have adverse effects on the environment, food web and human health^25^. In contrast, the production of these metal oxide nanoparticles (NPs) by green synthesis eliminates their adverse effects, gives them different properties and makes them widely used in many different fields. On the other hand, the consumption of various metal nanoparticles causes lipid peroxidation and mitochondrial damage producing reactive oxygen molecules. Therefore, in order to expand the use of metal and metal-based NPs in many fields, a thorough investigation of the harmful effects of metal nanoparticles on the environment and organisms is necessary. Therefore, it has been reported that a different protective coating on metal oxide NPs made of natural or macromolecular material can reduce the negative effects on the human body^25^.

Nickel oxide (NiONPs) nanoparticles with advanced nanoscale properties are used in photocatalysis, drug delivery, catalysis, semiconductors, micro-supercapacitors, tuned circuits, thermistors, solar cells, etc^26^. NiONPs have enhanced behavior and properties due to surface, volume, quantum size and microscopic tunneling effects. They are suitable for electrostatic immobilization of proteins with low ionization potential. Their physical and chemical properties depend on their morphology and particle size. NiONPs have been extensively studied due to their lower melting point compared to bulky forms, high mechanical strength, chemical stability and electron transfer capabilities^27^. In recent years, nickel oxide nanoparticles (NiONPs) have been prepared by green synthesis technique in an environmentally friendly approach using different lice species, enzymes and fungi and both cytotoxicity and antibacterial properties have been investigated^28–31^. In particular, plant extracts are widely used in green synthesis because they are readily available. The content of the plants used in the production of nanoparticles, the structure and texture of the leaves act as capping agents during nanoparticle production, trapping the metal ion at appropriate sites and having the ability to control the size of the nanoparticle^32^. Moringa oleifera which is a species of tree found in South and Southeast Asia and belongs to the Moringaceae family has been used in this present study to production NiONPs^33^. It grows rapidly and is often referred to as the drumstick tree. It has a high medicinal value in traditional herbal medicine^26^ and its leaves, flowers and pods can be used as a nutritious vegetable in Indian cuisine. The leaves also contain high levels of vitamin C, polysaccharides, secondary metabolites such as flavonoids and phenolic acids, amino acids and minerals with antibacterial and antioxidant properties^34^.

A review of the literature reveals a limited number of studies on the mechanisms of action of NiONPs prepared using the Moringa oleifera plant as a biomaterial^35^. In particular, there is a lack of studies on the cytotoxic effect, antibacterial activity and biocompatibility of these nanoparticles against HEP-G2 (a human liver cancer cell line). Therefore, in this study, NiONPs were prepared by green synthesis method using Moringa oleifera leaves. The high cytotoxic effect of the obtained NiONPs against HEP-G2 (a human liver cancer cell line), antibacterial activity against bacterial pathogens, morphology, structural and optical properties were investigated.

## Experimental method

### Process of making Moringa Oleifera extract

The Moringa Oleifera leaves used in this study were collected in November 2023 from the National Institute of Technology (NIT) campus in Rourkela, Odisha, India. The leaves were then rinsed with a solution of sterile distilled water, following which they were chopped into small pieces and ground using a mortar. A quantity of 20 g of leaves was meticulously combined with 100 ml of distilled water and ethanol. The amalgam was then subjected to a 24-hour period of ambient temperature storage. Following the incubation period, Whitman No. 1 filter paper was utilized for the comprehensive filtration of the resultant infusion, which was subsequently examined for antibacterial and qualitative cytotoxic properties.

### Synthesis of NiONPs

**Fig. 1:**
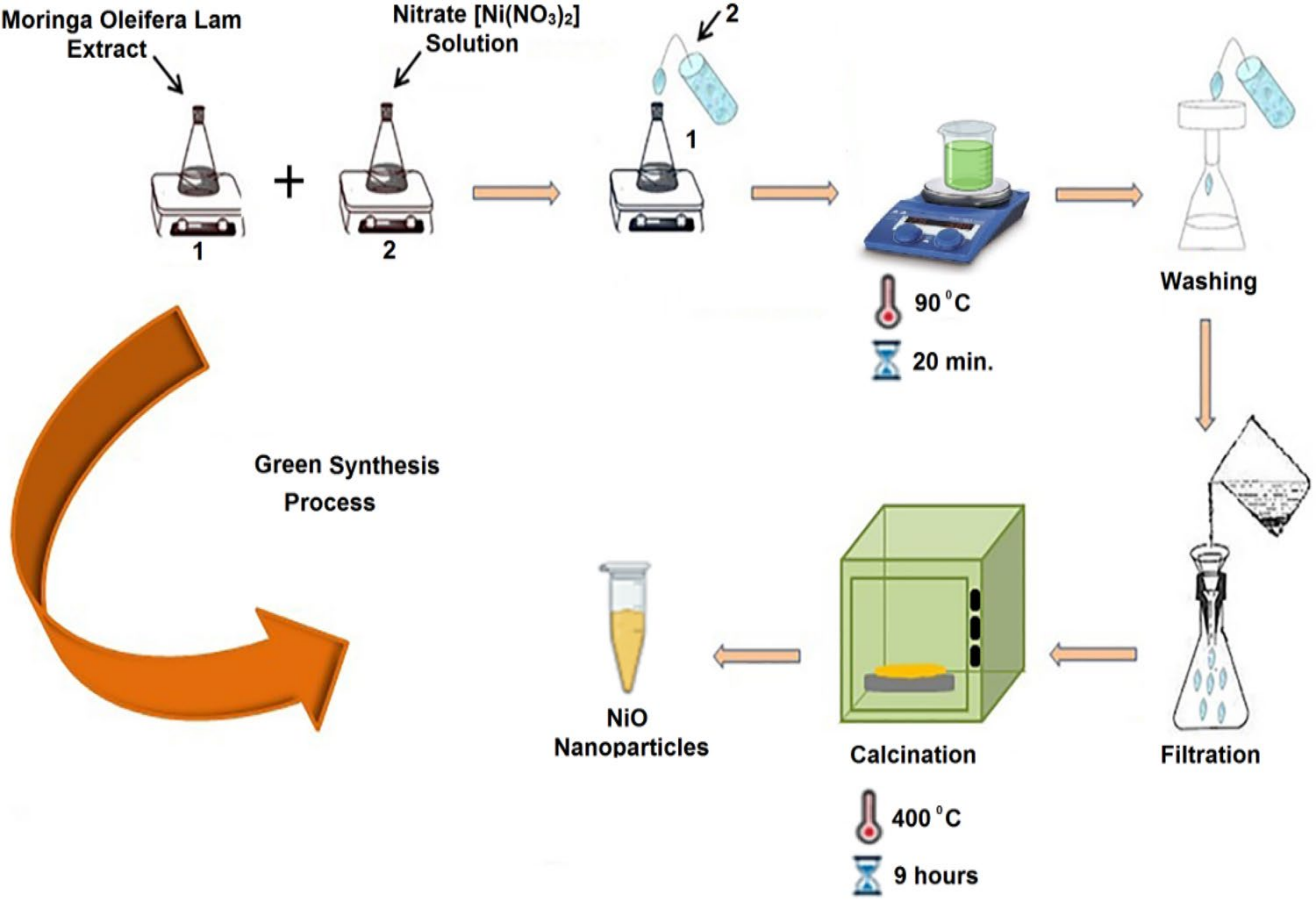
Green synthesis process of NiONPs.

Nickel nanoparticles (NiONPs) were synthesized using Moringa Oleifera plant extract by the green synthesis method, as illustrated in Fig. 1. A total of 20 ml of Moringa Oleifera plant extract was mixed with 80 ml of 0.1 mM nickel(II) nitrate hexahydrate (Ni(NO₃)₂·6 H₂O) (99% (Sigma-Aldrich)) chemical salt. The mixture was then subjected to magnetic stirring at a temperature of 90 °C for duration of 20 min. Following the occurrence of a color change, the solution was washed and filtered through filter paper. The resultant mixture was then subjected to heat treatment at 400 °C for a period of 9 h, yielding NiONPs in the form of a light yellow powder. The bioreduction and stabilization process used in the environmentally friendly synthesis of nickel oxide nanoparticles (NiONPs) from plant extract is mainly powered by the phytochemicals found in the extract. Bioactive substances like flavonoids, phenols, terpenoids, aldehydes, and carboxylic acids were detected in the plant extract by GC-MS analysis. During the creation of nanoparticles, these phytochemicals serve as capping and reducing agents.

First, the phytochemicals in the extract interact with nickel ions (Ni^+ 2^) from nickel nitrate. Ni²⁺ is reduced to Ni⁰ by reducing agents such flavonoids and phenolic substances, which contribute electrons. The oxidation of NiO to NiONPs is facilitated by a subsequent controlled thermal treatment or calcination. By creating a phytochemical-based capping layer, the extract’s terpenoids and organic acids stabilize the developing nanoparticles and stop them from aggregating. For instance, substances with functional groups (-OH, -COOH, -CHO) that can chelate metal ions and encourage uniform nucleation and development of NiO nanoparticles include 5-hydroxymethylfurfural, eugenol, and catechol, which are commonly detected in plant extracts using GC-MS. Through regulated nucleation pathways, the presence of these functional groups aids in the creation of crystalline or spherical nanostructures.

Therefore, the three steps of the synthesis mechanism are as follows: (1) complexation of Ni2^+^ with phytochemicals, (2) bioreduction to Ni^2+^, and (3) thermal oxidation to NiO NPs, with simultaneous stabilization by extract-based capping agents. In addition to promoting repeatable green synthesis, an understanding of this mechanism emphasizes the importance of particular phytochemicals in influencing the physicochemical characteristics of the nanoparticles.

### Characterization of NiONPs

The size distribution and morphology of NiONPs were determined by means of a Transmission Electron Microscope (TEM) model Hitachi H-7650 from Germany. A thorough optical examination was subsequently conducted over a broad wavelength range (200–900 nm), encompassing the measurement of UV-visible absorption spectra. This investigation was facilitated by the utilization of a UVIR-210 A Shimadzu spectrophotometer, a sophisticated instrument that enabled precise and comprehensive analysis. Finally, the formation of nickel oxide nanoparticles was confirmed through the documentation of FTIR spectra with 500–4000 cm^− 1^ dispersion. The structural characteristics of the sample were analyzed using the X-ray diffractometer method (XRD, PW 1730, PHILIPS Co.). The radiation source employed in this study is a (Cu-Kα) source, with a wavelength of 1.54060 Å and a scanning angle (*2θ*) ranging from 10 to 80 degrees.

### In-vitro cytotoxic activity NiO nanoparticles by MTT assay

In order to investigate the biological effects of NiONPs, an in vitro cytotoxic activity assay was conducted using MTT. In this assay, HEP-G2 and MCF-7 cells were seeded in 96-well plates and treated in colloidal solutions for 24 h at 37 °C, following similar cytotoxicity protocols used in prior laser-based gold nanoparticle research on MCF-7 cancer cells^18^. The MTT assay was utilized to ascertain the percentage of cell death, employing a microtiter plate reader for data analysis. The statistical calculations were performed independently for each effect, and subsequently, the cumulative effect was determined by combining all effects. The 3-(4,5-dimethyl-2-thiazolyl)-2,5-diphenyl-tetrazolium bromide (MTT) test was utilized to ascertain the mitochondrial activity of the cultured cells. In order to evaluate the degree of toxicity, various extracts were added to the culture medium at varying concentrations of the NiO nanomaterial produced: The concentrations of the substance under investigation were 1000, 500, 250, 125, 62.5, 31.2, 15.6 and 7.8 µg/mL. The mitochondrial succinate dehydrogenase enzymes present within the living cells then converted the yellow dye MTT into purple formazan crystals. In order to achieve complete resolution, it was necessary to dissolve the crystals in DMSO. Subsequently, the optical density at various concentrations was measured using a spectrophotometer operating at a specific wavelength.

### Preparations of bacterial inoculum

The bacterial isolates S. aureus and E. coli were cultivated in Tryptone Soy Broth (TSB). The recommended dosage is 10 milliliters of purified water mixed with 0.9 g of TSB. Subsequent to this, both components are amalgamated with a stirrer, following which they are placed in an autoclave for a period of ten minutes. Colonies of S. aureus and E. coli bacteria are added to each TSB, and they are then incubated for a period of 24 h.

### Antimicrobial activity

The antibacterial effectiveness of NiONPs was explored against a range of human pathogenic bacteria, including two Gram-positive bacteria (*S. mutans*) and two Gram-negative bacteria (*E. coli*)^19^. The antibacterial activity was evaluated in accordance with the Clinical and Laboratory Standards Institute (CLSI) approach^34^. The target bacteria and NiONPs were examined for antimicrobial susceptibility using a disc diffusion experiment. Triplicates were created using various dilutions of sterile and deionized water. Following a 15-minute incubation period at ambient temperature, the isolates were subjected to an overnight incubation at 33 °C. The initial utilization of this document is to provide an explanation of technical word abbreviations. Positive results were reported when an inhibition zone was perceived surrounding the well after a period of induction, and the width of the inhibition zone was evaluated^20^.

## Results and discussion

### XRD analysis

**Fig. 2:**
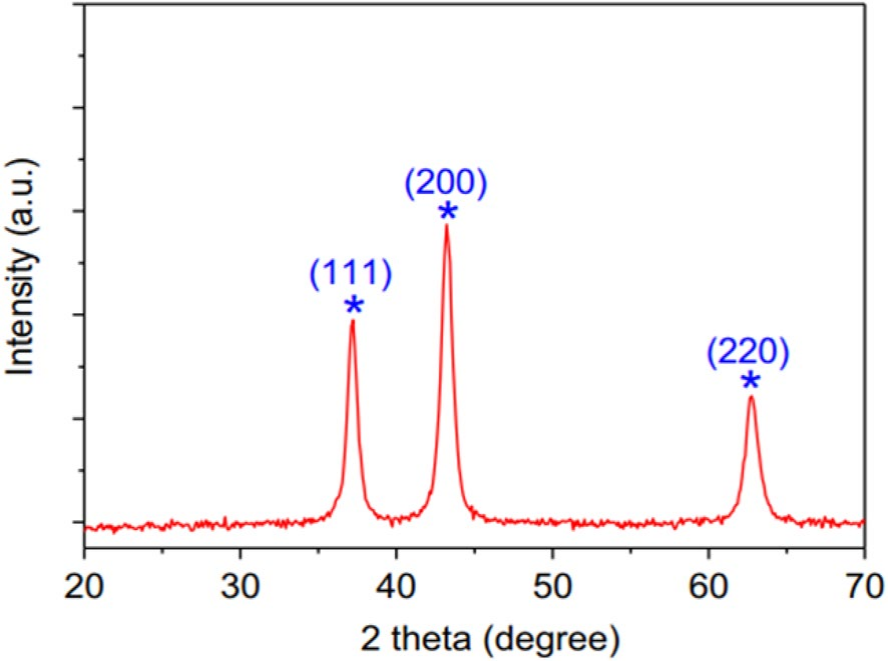
XRD pattern of NiONPs.

Figure 2 presents the X-ray diffraction (XRD) pattern of NiONPs. As demonstrated by the diffraction patterns, NiO NPs formed three strong and sharp peaks at diffraction angles of 37.2°, 43.2°, and 62.8°. The presence of NiO (JCPDS file no. 4-835) is indicated by these crystal planes. The diffraction peaks observed at these angles are attributed to the 111, 200, 220 planes, respectively. The findings demonstrate that NiONPs exhibit growth predominantly in the 111, 200, 220 directions when produced by laser ablation technique. Furthermore, the intensity and half-peak width of these peaks are indicative of the level of crystallization, and it can be posited that the produced NiONPs demonstrate good crystallization due to the sharp peak intensities and small half-peak widths. In the XRD spectrum, no peak belonging to another phase was observed, with the exception of the three NiO peaks. Consequently, it was determined that the NiONPs produced are pure and do not contain any contaminants or by-products other than NiONPs. The structural parameters of NiONPs were the focus of the study, and comprehensive data on these was obtained through calculations of the grain size (D). The grain size values were checked using the Scherrer formula^14^:

1 $$\:D=\frac{K\lambda}{\beta\:cos\:\theta}$$

2 $$\delta=\frac{1}{{D}^{2}}$$

where is where *λ* is the wavelength of *CuK*_*α*_ light (with a value of 1.5406 Å), *β* is the peak at half maximum (FWHM) width in radians, and *θ* is the diffraction angle, Table 1 shows the diffraction angles (*2θ*), Miller indices (*hkl*), half peak widths (FWHM) and grain size (*D*), and dislocation density (*δ*) values for the peaks observed in the XRD patterns of NiONPs. The findings suggest a relationship between the plane of growth and the grain size values of NiONPs, indicating potential variations across different growth surfaces. The average grain size of NiONPs produced was determined to be 11.2, 9.2 and 6.6 nm for growth in the dominant (111), (200) and (220) directions, respectively. It was observed that NiONPs of different sizes were formed in different orientations. NiONPs formation occurs in different orientations and directions, leading to variations in dislocation density. The smallest grain sizes and dislocation densities are observed in growth along the (220) and (111) directions, respectively.

**Table 1:** The structural parameters of NiONP_S_.

2θ (Deg.)	FWHM (Deg.)	d_hkl_ Exp.(Å)	D (nm)	d_hkl_ Std.(Å)	hkl	δ (nm^− 1^)	Crystal System	No.
37.2270	0.7465	2.4134	11.2	2.3689	(111)	8.2 × 10^− 2^	Cubic	4-835
43.2000	0.9331	2.0925	9.2	2.0515	(200)	1.08 × 10^− 1^	Cubic	4-835
62.2000	1.3997	1.4913	6.6	1.4506	(220)	1.51 × 10^− 1^	Cubic	4-835

### FESEM and fourier transform infrared spectroscopy (FTIR) analysis

**Fig. 3:**
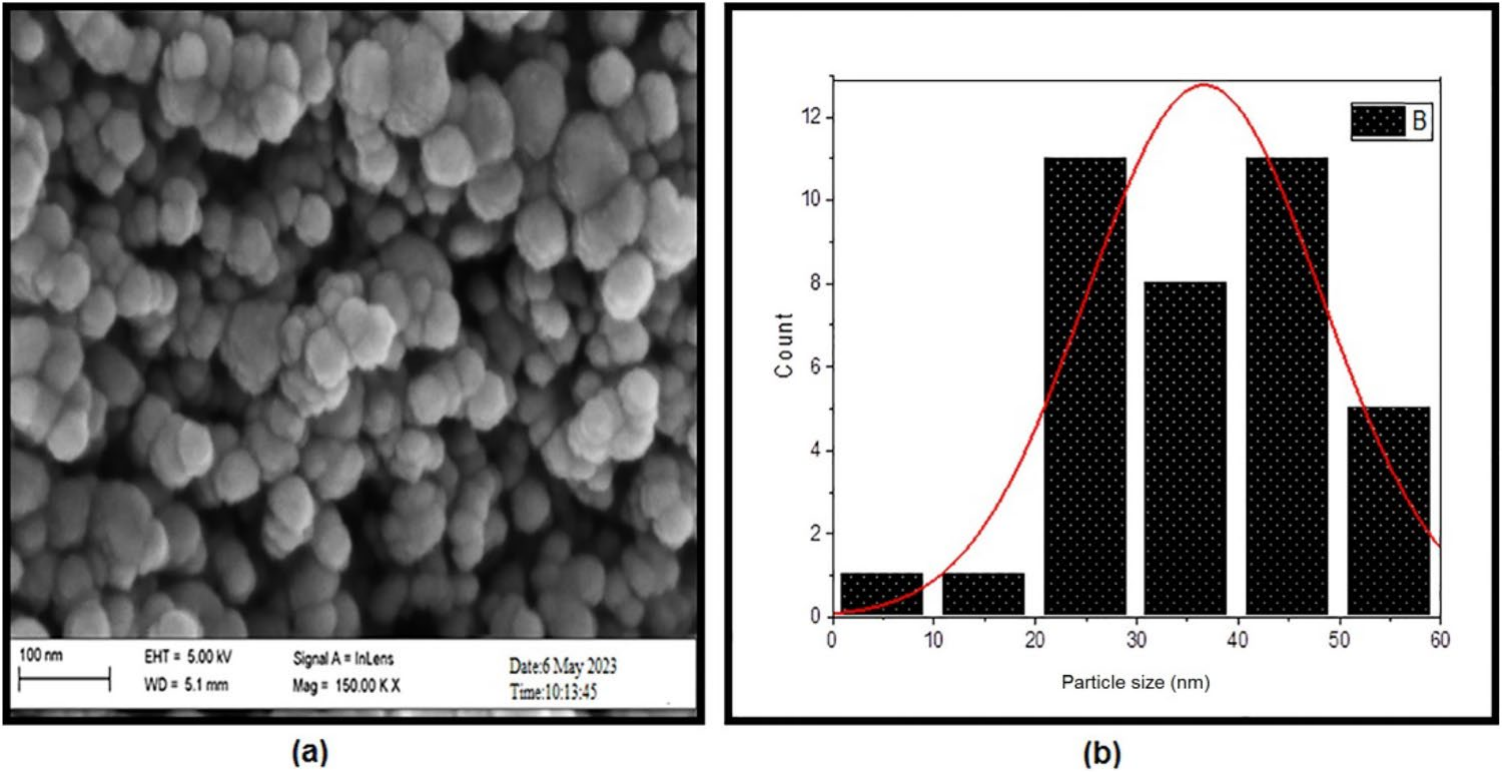
FESEM image of NiONPs.

Figure 3 (a) and (b) illustrate FESEM images, particle distributions and particle sizes of NiONPs. The images demonstrate that the morphological structure of NiONPs is both spherical and homogeneous. The SEM image reveals a particle size distribution characterized by spherical surface topography over a diameter range of (29–43) nm.

**Fig. 4:**
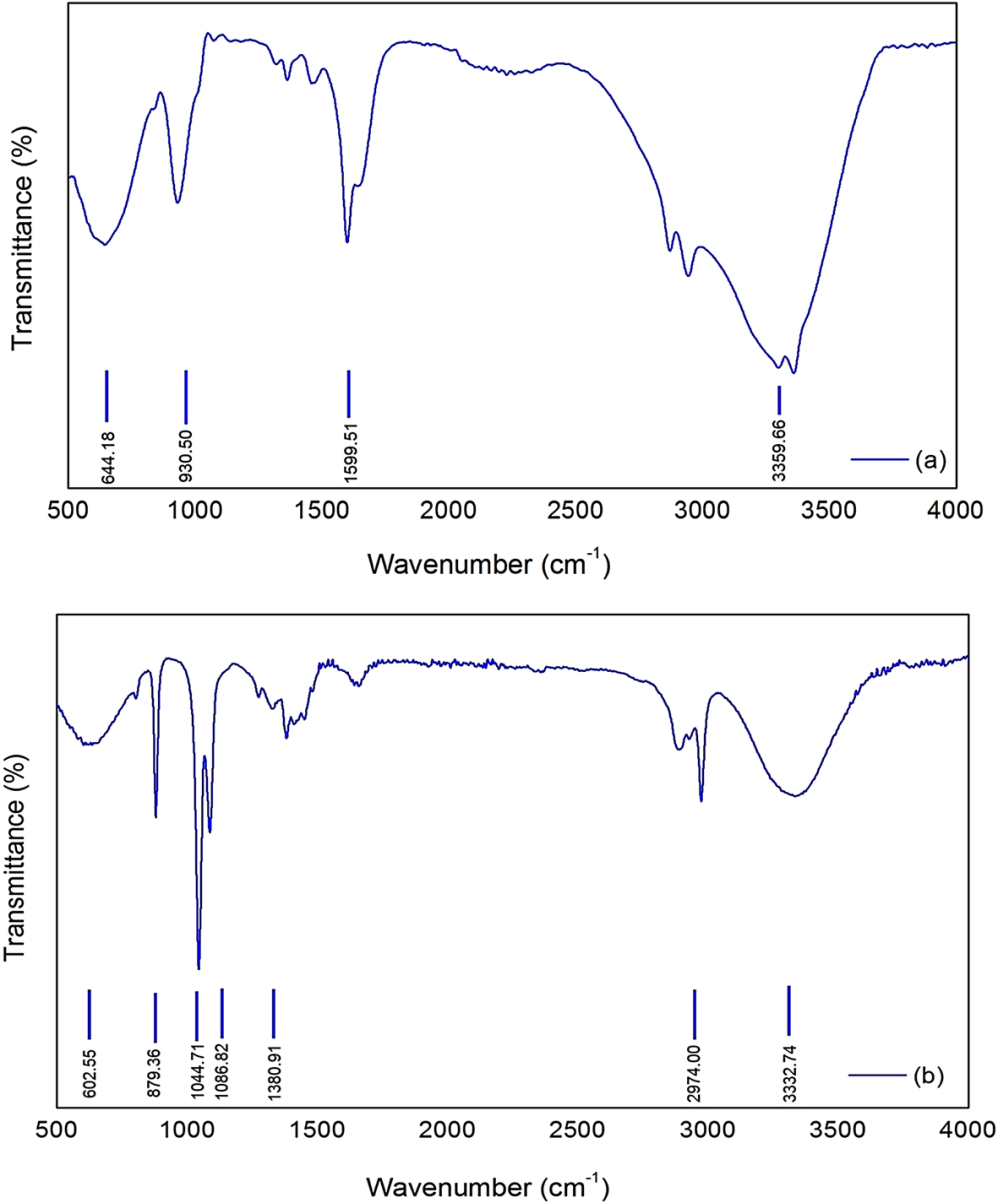
FTIR spectra of (**a**) NiONPs and (**b**) *M. oleifera* leave extract.

To facilitate a comprehensive understanding of the functional group present within the structural configuration of NiONPs, the FTIR spectra underwent thorough characterization over the wavelength range from 400 to 900 nm. The FTIR spectra of NiOPs are displayed in Fig. 4. As displayed in Fig. 4(a), the FTIR spectrum of the plant extract exhibits O-H stretching vibrations at 3300 cm^− 1^, C = C or C = O stretching vibrations at 1600 cm^− 1^, and C-O stretching vibrations at 1050 cm^− 1^. The presence of phytochemicals such as alcohols, flavonoids and phenolic compounds is confirmed by these peaks. The spectrum exhibited a band in the range from 400 to 900 cm, attributed to the presence of Ni-O-Ni bonds, in addition to the potential presence of Ni-O-C bonds. The observed peaks at 1087.12 and 879.83 cm^− 1^ indicated the formation of a metal-oxide (Ni-O) bond, thereby confirming the configuration of the NiONPs^36,41^. The intensity of the O-H peaks is lower in comparison to the pure nickel oxide peaks. Conversely, a discernible decline in the intensity of O-H and C = O stretching peaks was evident in the FTIR spectra of the NiO nanoparticles produced, indicating the potential involvement of these functional groups in the nanoparticle formation process. The hypothesis that the efficient synthesis of NiO nanoparticles was confirmed by the appearance of a new peak at approximately 550 cm^− 1^, attributed to the Ni-O bond, was further substantiated. This finding indicates that the conversion of NiO to oxide NiO was reduced and that the NiONPs complex was successfully prepared^21,22^.

### Surface plasmon resonance of NiONPs

**Fig. 5:**
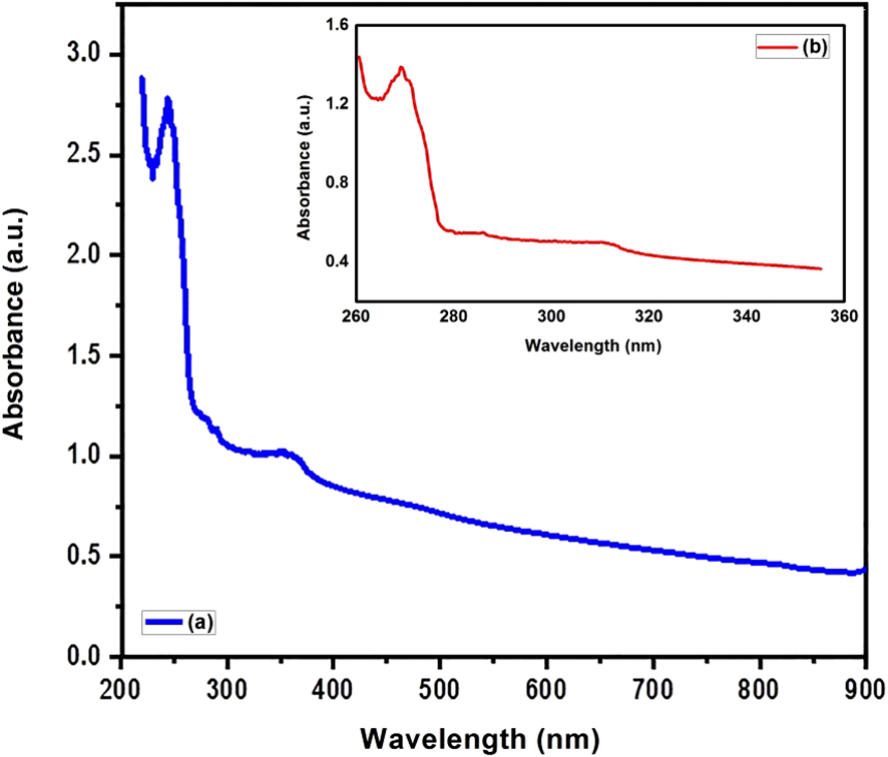
The absorbance spectra of (**a**) NiONPs and (**b**) *M. oleifera* leaves extract.

The Fig. 5 shows the absorbance spectra of NiONPs and *M. oleifera* leaves extract across the wavelength range of 200–900 nm. It was determined that NiONPs obtained from *M. oleifera* plants exhibit surface plasmon resonance, and a comparison was made with a previous report. According to Fig. 5 (b); the UV-Vis absorption spectra of the *M. oleifera* leaves extract exhibited distinct absorption bands within the 260–290 nm range. These peaks are indicative of π→π* transitions in aromatic rings and n→π* transitions in carbonyl groups, suggesting the presence of phenolic chemicals and flavonoids. Following the synthesis of NiO nanoparticles in the presence of a plant extract, a significant absorption peak within the 250–360 nm regions was identified. The observation of a red shift in the absorption spectrum relative to the plant extract indicates the successful formation of NiO nanoparticles. The observed peak is attributed to interband electronic transitions and probable surface plasmon resonance (SPR) phenomena. Also; the obtained NPs exhibited the characteristic surface plasmon resonance (SPR) band within the spectral range of 250–356 nm (Fig. 5(a)). The formation of NiONPs was confirmed by the observation of a band at the wavelength of 356 nm^16,37,38,41^. Consequently, the SPR peak can be employed not only to ascertain the particle size for a UV–vis spectrum below 600 nm, but also to determine the shape of the particle^24^.

### **Methods cell lines and culture**

The human liver cancer cell line known as HEP-G2 was procured from the National Cell Bank of Iran, which is part of the Pasteur Institute in Iran. The cells were cultivated in RPMI-1640 (Gibco) with 10% Foetal Bovine Serum (FBS, Gibco), with the addition of antibiotics (100 U/ml penicillin and 100 µg/ml streptomycin). The maintenance of cells was conducted at a temperature of 37 °C, within an environment containing humidified air and 5% CO₂. The passage of cells was facilitated through the utilisation of 1X trypsin/EDTA (Gibco) and a phosphate-buffered saline (PBS) solution.

MTT cell viability assay. The quantification of cell growth and cell viability was conducted using the MTT [3-(4, 5-dimethylthiazol-2-yl)-2, 5-diphenyltetrazolium bromide] (Sigma-Aldrich) assay. In summary, the cells were digested with trypsin/EDTA, harvested, and adjusted to a density of 1.4 × 10⁴ cells/well. Thereafter, the cells were seeded into 96-well plates, which were filled with 200 µl of fresh medium per well, and left undisturbed for 24 h. Once a monolayer had formed, the cells were treated with a range of concentrations (200–12.5 µg/ml) of the compounds, in a DMSO solution, for a period of 24 h at 37 °C in 5% CO₂. Following the conclusion of the treatment period (24 h), while the monolayer culture was left untouched in its original plate, the culture medium was removed and 200 µl/well of MTT solution (0.5 mg/ml in phosphate-buffered saline [PBS]) was added. The plate was then incubated at 37 °C for an additional 4 h.

MTT solution: the culture medium was removed and dimethyl sulfoxide was added (100 µl per well). The cells were then subjected to an incubation process on a shaker at a temperature of 37 °C, with the objective of achieving complete dissolution of the crystals. The quantification of cell viability was conducted by measuring the optical density at a wavelength of 570 nm, employing an ELISA reader (Model Wave xs2, BioTek, USA). The concentration of the compounds that resulted in 50% of cell death (IC50) was determined from respective dose-response curves.

### In vitro cytotoxicity studies

Utilizing the MTT test paradigm, the cytotoxic properties of the produced nickel oxide nanoparticles were investigated. The investigation of morphological damage or inhibition of the zone of outgrowth caused by the drugs under test forms part of the process of determining their cytotoxic properties. The CO_2_ incubator (WTC Binder, Germany) and ELISA reader (for MTP) (Anthos 2010, Germany) were utilized in the research.

MTT Assay for Anti-Proliferative Activity: MTT is a fluorometric assay that quantifies the metabolic activity of living cells. The complete experiment may be carried out in a micro titer plate (MTP) and is non-radioactive. The application has been shown to be effective in the calculation of parameters such as cellular toxicity, cell viability, and proliferation. The fundamental principle of this procedure is that living cells convert MTT into the insoluble formazan salt. The substance is both quantifiable and soluble. An increase in the concentration of viable cells is indicative of a higher quantity. The number of cells under investigation is closely correlated with the corresponding value of the absorbance. This technique has been demonstrated to be effective in the cultivation of adherent cells in MTP, as well as in the cultivation of normal human dermal fibroblasts (NHDFs).

The present study evaluated the cytotoxic potential of nickel oxide nanoparticles (NiONPs) at varying concentrations and doses by employing the MTT test technique. The results demonstrated a direct correlation between the concentration of NiONPs and their lethal activity, with higher concentrations resulting in increased toxicity. Figure 1 clearly shows that the NiONPs used in this study were cytotoxic to human cancer cells, with an optimal percentage of cell death of 91.05% achieved at a concentration of 100 µg/ml.

**Fig. 6:**
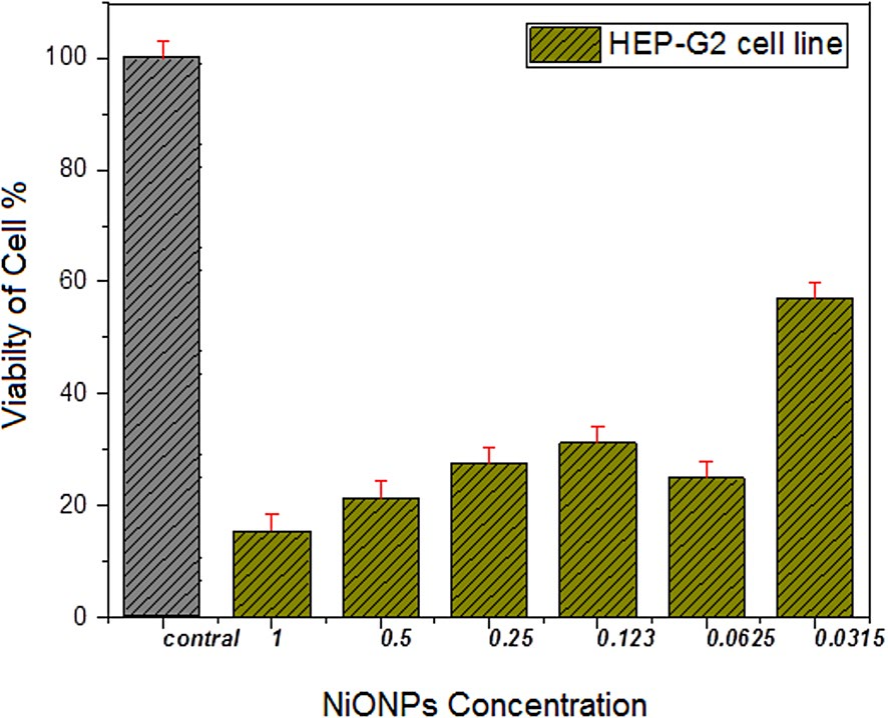
The effect of the combination of NiONPs on HEP-G2 cell line.

**Fig. 7:**
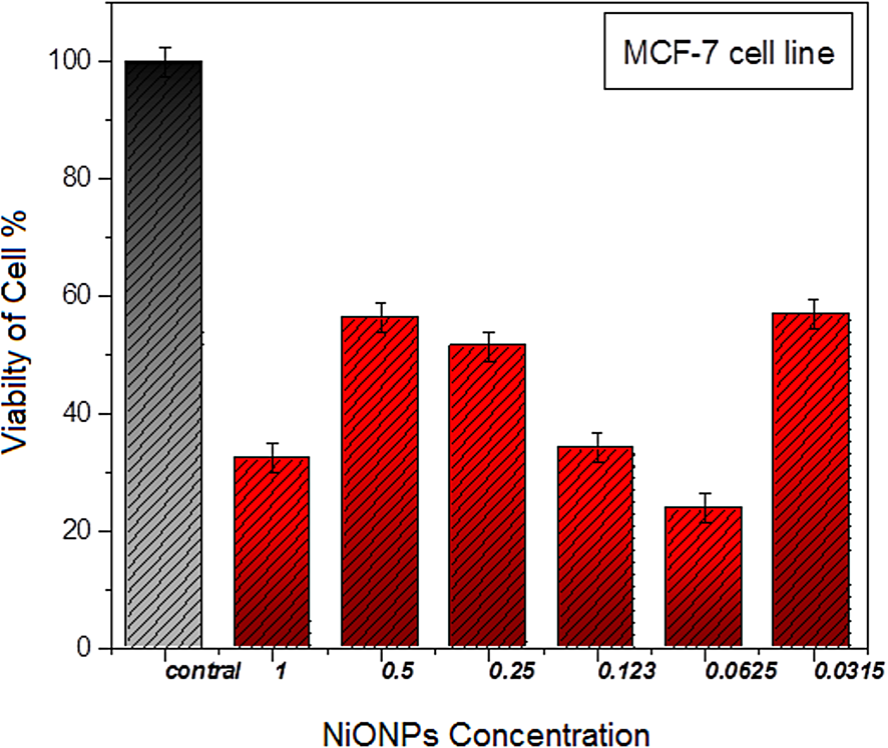
The effect of the combination of NiONPs on MCF-7 cell line.

As depicted in Fig. 6, six distinct concentrations were examined: 1, 0.5, 0.25, 0.123, 0.0625, and 0.0315 µg/ml. The data indicate that the concentration of 1 µg/ml exerts a significant impact on the viability of HEP-G2 cells, resulting in a 85.2% mortality rate, while the remaining cells (15.40%) exhibited survival characteristics. As demonstrated in Fig. 7, the concentration of 0.0625 µg/ml has been shown to exert a significant impact on the viability of the MCF-7 cell line, resulting in a 76.10% mortality rate, while the percentage of cells that survived was recorded at 23.90%.

### Antimicrobial activity of NiONPs

**Fig. 8:**
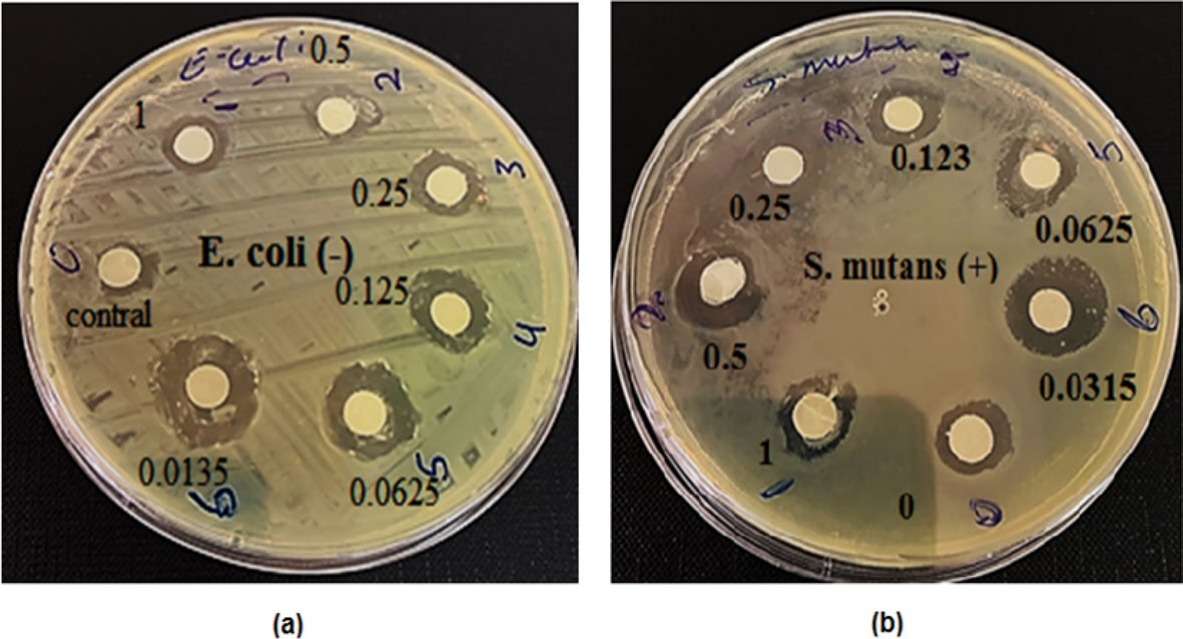
Inhibition zone *of E. coli* and *S. mutans* bacterial growth as a result of antibacterial activity of NiONPs.

**Fig. 9:**
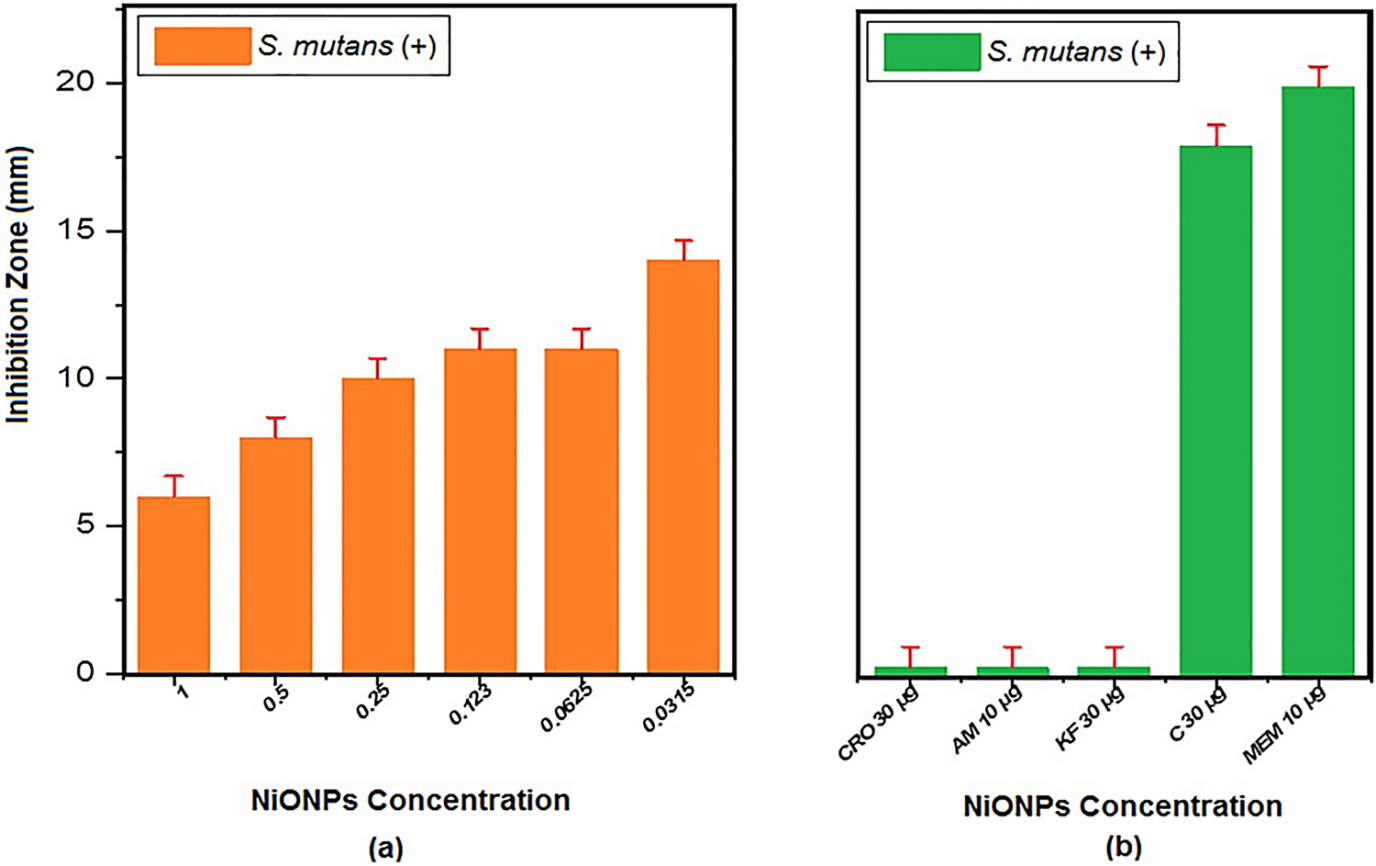
(**a**) Antibacterial action on *S. mutans* (+) (**b**) Inhibition zone diameter of antibiotics of NiONPs Concentration.

**Fig. 10:**
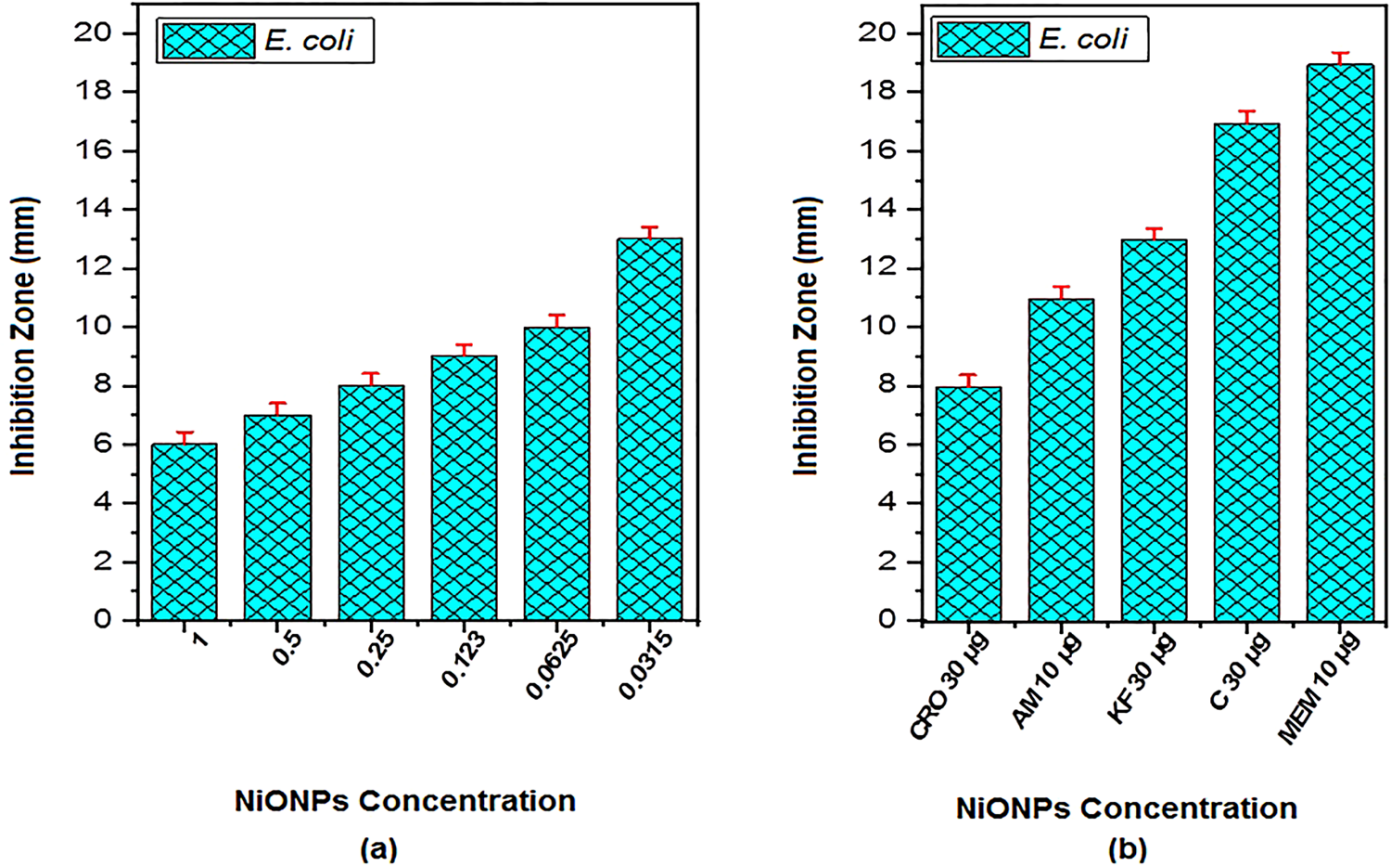
(**a**) Antibacterial action on *E. Coli* (**b**) Inhibition zone diameter of antibiotics of NiONPs concentration.

The antimicrobial activity of NiONPs was tested against bacterial isolates maintained on nutrient agar slants, following the Clinical and Laboratory Standards Institute (CLSI) guidelines. NiONPs were prepared at different concentrations (1, 0.5, 0.25, 0.123, 0.0625, and 0.0315 μg/mL) diluted in sterile deionized water. The bacterial isolates were incubated with NiONPs for 15 min at room temperature, then incubated overnight at 37 °C. Positive antibacterial activity was confirmed by the presence of inhibition zones around wells after incubation. The diameters of inhibition zones were measured in millimeters (mm) using a digital Vernier caliper. As shown in Fig. 8; Tables 2 and 3, there was a significant relationship between nanoparticle concentration and inhibition zone size (*P* < 0.05). Notably, the inhibition zones increased as the concentration decreased, with the largest zones observed at 0.0315 μg/mL, exceeding some standard antibiotics. Gram-positive bacteria (S. mutans) displayed somewhat bigger inhibitory zones than Gram-negative bacteria (E. coli) at the same nanoparticle doses in the published antibacterial investigation of NiO nanoparticles (NiONPs). For instance, the inhibition zone was 14 mm for S. mutans and 13 mm for E. coli at the lowest dosage of 0.0315 µg/mL (Figs. 9 and 10).

The maximum inhibition zone diameters recorded were 13.0 ± 0.5 mm against Escherichia coli and 14.0 ± 0.6 mm against Streptococcus mutans. The antibacterial effect is attributed to reactive oxygen species (ROS) generation causing oxidative stress and damage to bacterial proteins and DNA, as well as electrostatic interactions disrupting bacterial cell membranes. Reactive oxygen species (ROS) produced by NiONPs cause oxidative stress, which harms the proteins, DNA, and cell membranes of bacteria. Bacterial mortality results from membrane breakdown and intracellular content leaking brought on by electrostatic interactions between positively charged NiONPs and negatively charged bacterial cell walls^42^.

**Table 2:** Antibacterial activity of NiONPs against some pathogenic bacteria isolates.

Bacterial isolates	Inhibition Zone Diameter of Antibiotics (mm)				
	CRO 30 µg	AM 10 µg	KF 30 µg	C 30 µg	MEM 10 µg
*E. coli* (-)	8	11	13	17	19
*S. mutans* (+)	0	0	0	18	20

**Table 3:** Antibacterial activity of NiONPs against some pathogenic bacteria isolates (inhibition zone diameter in mm) compared with antibiotics under study.

Bacterial Isolate	Concentration (µg/mL)	Replicate 1 (mm)	Replicate 2 (mm)	Replicate 3 (mm)	Mean (mm)	Standard Deviation (mm)
E. coli (-)	1	6.1	5.9	6.0	6.0	0.10
	0.5	7.2	6.8	7.0	7.0	0.20
	0.25	8.0	8.5	8.0	8.17	0.29
	0.123	9.1	8.9	9.0	9.0	0.10
	0.0625	10.1	9.9	10.0	10.0	0.10
	0.0315	13.0	12.7	13.0	12.9	0.17
S. mutans (+)	1	6.1	5.9	6.0	6.0	0.10
	0.5	8.0	7.8	8.0	7.93	0.12
	0.25	10.0	9.8	10.0	9.93	0.12
	0.123	11.2	10.9	11.0	11.03	0.15
	0.0625	11.3	9.9	11.2	10.8	0.77
	0.0315	14.1	13.7	14.0	13.93	0.23

## Conclusion

The aim of the present study was to produce non-toxic, economical and environmentally friendly NiO nanoparticles with biomedical applications. Synthesis of NiO nanoparticles was therefore carried out using *M. Oleifera* extract, a medicinal plant that has been used in the production of cytotoxic agents with a view to preventing the spread of cancer cells. The formation of NiO nanoparticles was confirmed by analytical techniques such as UV-visible, XRD, FTIR, and SEM analyses. In the UV-visible spectrum, the SPR band was formed at 356 nm. The XRD pattern revealed that the nanoparticles have a cubic crystal structure, high crystallinity and an average crystallite size of 9 nm. The metal-oxygen bond in the nanoparticles was understood from the vibration bands at 1087.12 and 879.83 cm^− 1^ in the FTIR spectrum. NiONPs demonstrated a high level of toxicity, with a percentage of 91.05% being observed in HEP-G2 cancer cells. At a concentration of 1 µg/ml, the substance demonstrated a significant impact on the viability of HEP-G2 cells, resulting in the demise of 85.2% of them. Similarly, at a low concentration of 0.0625 µg/ml, the substance exhibited a notable effect on the viability of the MCF-7 cell line, leading to the destruction of 76.10% of them. The investigation established that NiO nanoparticles exhibited high levels of antibiotic sensitivity and antibacterial activity in the presence of Chloramphenicol (C) and Meropenem (MEM) antibiotics, as well as *S. mutans* and E. *coli* bacteria, respectively. The highest antibiotic susceptibility was demonstrated to Chloramphenicol (C) and Meropenem (MEM) antibiotics. The highest levels of antibacterial resistance were demonstrated at low concentrations (0.0315 µg/mol). The results indicate that NiONPs prepared with Moringa oleifera plants exhibit non-carcinogenic properties, high biocompatibility, and effective drug activity. Consequently, it possesses the potential to be utilized in medical applications for the treatment of various diseases.

## Data Availability

All the data are presented in this manuscript.

## Declarations

### Guidelines for plants

The authors attest that the current study’s use of plants conforms with institutional, national, and/or international regulations.

**Permissions to collect** the plants/plant parts all plant specimens collected with permissions.

**Source of the plant** used in your study all plants seeds name and he source of them are in the Methods section.
